# Supplementary material for: Identification of photoperiod-induced specific miRNAs in the adrenal glands of Sunite sheep (Ovis aries)
Source: Front Vet Sci. 2022 Jul 22;9:888207. doi: 10.3389/fvets.2022.888207 (PMC9354845; doi:10.3389/fvets.2022.888207)
Supplement: Supplementary file 1 [file Data_Sheet_1.ZIP › Supplementary file/miRDeep2 file.zip!/miRDeep2 file/Know_Structure/SPLP42A2/oar-mir-370.pdf]

miRBase precursor : oar-mir-370
Total read count : 9961
oar-miR-370-5p read count : 49
oar-miR-370-3p read count : 9900
remaining reads : 12

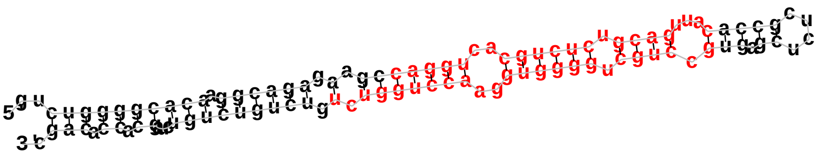

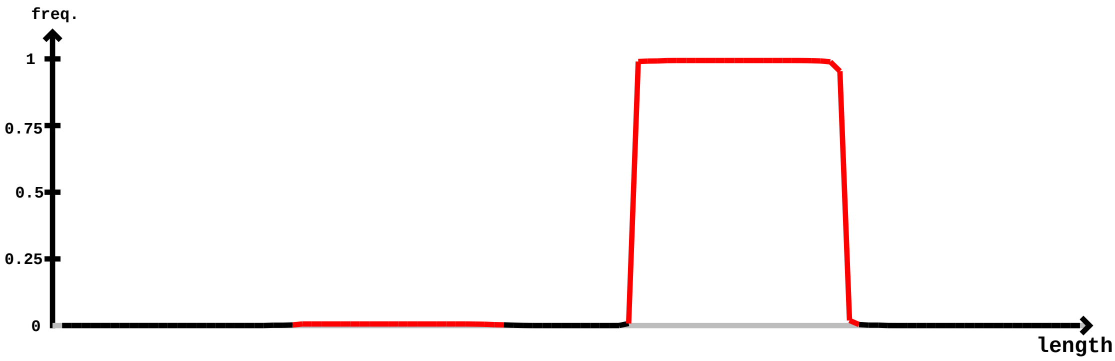

oar-miR-370-3p

oar-miR-370-5p

5'- gucuggggcacaaggcagagaagccaggucacgucucugcaguuacaccgcucucgagugccugcugggguggaaccuggucugucugucugucuagcaccacagc -3' exp

..((((((((((.((((((.(.(((((((..((((((.((((...(((((....)).))).)))).))))))..))))))).).)))))))))...)).)).))).	reads	mm	sample
gucuggggcacaaggcagagaagc..	1	0	seq
.....................agccaggucacgucucugcag..	4	0	seq
.....................agccaggucacgucucugcagu...	6	0	seq
.....................agccaggucacgucucugcaguu..	1	0	seq
.......................ccaggucacgucucugcagu...	1	0	seq
.......................ccaggucacgucucugcaguu..	9	0	seq
.......................ccaggucacgucucugcaguua...	1	0	seq
........................caggucacgucucugcaguu..	5	0	seq
........................caggucacgucucugcaguua...	7	0	seq
........................caggucacgucucugcaguuac..	13	0	seq
........................caggucacgucucugcaguuaca...	9	0	seq
........................caggucacgucucugcaguuacac..	4	0	seq
....gugccugcugggguggaaccuggu.........................	1	0	seq
....ugccugcugggguggaac..............................	1	0	seq
....ugccugcugggguggaacU.............................	1	1	seq
....ugccugcugggguggaaccug...........................	1	0	seq
....ugccugcugggguggaaccugg..........................	8	0	seq
....ugccugcugggguggaaccuggu.........................	63	0	seq
....ugccugcugggguggaaccugguc........................	3	0	seq
....gccugcugggguggaacc.............................	3	0	seq
....gccugcugggguggaacU.............................	1	1	seq
....gccugcugggguggaaccu............................	11	0	seq
....gccugcugggguggaaccug...........................	31	0	seq
....gccugcugggguggaaccugg..........................	340	0	seq
....gccugcugggguggaaccuggu.........................	9239	0	seq
....gccugcugggguggaaccugguc........................	140	0	seq
....gccugcugggguggaaccuggucu.......................	18	0	seq
....ccugcugggguggaaccuggu.........................	6	0	seq
....ccugcugggguggaaccugguc........................	8	0	seq
....ccugcugggguggaaccuggucu.......................	3	0	seq
....cugcugggguggaaccugg..........................	1	0	seq
....cugcugggguggaaccuggu.........................	2	0	seq
....cugcugggguggaaccugguc........................	1	0	seq
....cugcugggguggaaccuggucu.......................	1	0	seq

oar-miR-370-3p

oar-miR-370-5p

gucuggggcacaaggcagagaagccaggucacgucucugcaguuacaccgcucucgagugccugcugggguggaaccuggucugucugucugucuagcaccacagc

....ugcugggguggaaccugguc........................ 1 0 seq
....ugcugggguggaaccuggucug...................... 1 0 seq
....ugcugggguggaaccuggucugu..................... 12 0 seq
....ugcugggguggaaccuggucuguc.................... 1 0 seq
....gcugggguggaaccuggucugu..................... 2 0 seq
